# Mindfulness intervention for foundation year doctors: a feasibility study

**DOI:** 10.1186/s40814-019-0449-y

**Published:** 2019-04-27

**Authors:** Christopher Nyi Nyi Bu, Elizabeth Cotzias, Maria Panagioti

**Affiliations:** 1 Royal Liverpool University Hospital, Royal Liverpool and Broadgreen University Hospitals Trust, Prescot Street, Liverpool, L7 8XP UK; 2 Centre for Primary Care, Division of Population Health, Health Services Research and Primary Care, University of Manchester, Suite 4, Floor 6, Williamson Building, Oxford Road, Manchester, M13 9PL UK

**Keywords:** Mindfulness, Foundation, Junior doctor, Feasibility, Burnout

## Abstract

**Background:**

Mindfulness has been shown to reduce stress and burnout in medical students and healthcare professionals. This is a quality improvement study which assessed the feasibility of conducting a full-scale evaluation of a mindfulness intervention among UK foundation doctors to reduce stress and burnout.

**Methods:**

This is an uncontrolled before and after study taking place in a single university teaching hospital. The RE-AIM framework which comprises of five dimensions including Reach, Adoption, Effectiveness, Implementation, and Maintenance was used to guide this assessment. The intervention was a 6-week ‘Mindfulness in the Workplace’ course. The primary measure was change in self-reported levels of stress immediately before and after the course. Additional measures explored the subjective experiences of participating doctors through the use of questionnaires handed out before and after the course.

**Results:**

All 20 places on the course were filled from the population of 108 foundation doctors at the trust with an equal number of foundation year 1 (*n* = 10) and foundation year 2 (*n* = 10) doctors. Sixteen participants (80%) attended one or more sessions. The median baseline stress score of the participants was 6.5 (range = 2 to 9). The median post-course stress score was 5.0 (range = 2 to 8). The Mann-Witney test indicated that the stress levels of participants were significantly lower at the end of the course compared to baseline, *U* = 74.50, *p* = .04. Additional measures suggested that the intervention may be associated with some other potential promising benefits for doctors including greater wellbeing, improved working life, and more satisfactory relationships with patients. Implementation of this intervention requires further work at the institutional level because only 35% of participants completed the full intervention, the main barrier being work commitments.

**Conclusion:**

This is the first programme of research to evaluate the feasibility of trialling and implementing a modified ‘Mindfulness in the Workplace’ intervention for foundation junior doctors in the UK. Based on the findings from this study, we conclude that this intervention is promising but further modifications are required such as the use of validated outcome measures and improving delivery aspects before this intervention programme is trialled among foundation doctors in the UK.

## Background

The 2018 General Medical Council (GMC) national training survey found that almost a quarter of junior doctors in training experience burnout because of their work [1], and a previous survey suggested that first (F1) and second year (F2) junior doctors experience the lowest overall satisfaction [2]. There is evidence that doctors struggle to maintain a balance between having to perform as objective, competent medical clinicians whilst having to be sensitive, caring, and emotionally intelligent at the same time [3]. These interpersonal skills are championed by the GMC for all doctors [4] and in a sense comprise a form of emotional labour [3]. These demands become particularly evident for F1 doctors during the transition from student to doctor when they should be assisted with developing the coping skills they need [5]. Several studies have also demonstrated a link between burnout in doctors and reduced patient safety [6, 7]. Several studies suggest that medical students and trainees [8] report higher rates of depression than the general population [9] and are experiencing burnout even before they qualify as doctors [10]. Given the high rates of burnout and depression in trainee doctors and the observed links between doctor stress and patient safety in this transition period, there is a need to develop diverse approaches to support trainees [11].

One promising approach to support individual trainees is mindfulness. A useful definition of mindfulness is the skill of ‘learning to pay attention moment to moment, intentionally, and with curiosity and compassion’ [12]. Originating from eastern Buddhist meditation practice, mindfulness-based interventions are now a secular practice prevalent throughout the Western World. Nationally, an All-Party Parliamentary Group was formed in 2015 to develop the ‘Mindful Nation UK’ report, which recommends that mindfulness-based stress reduction courses such as mindfulness-based cognitive therapy (MBCT) should be implemented in education, the criminal justice system, the workplace, and in healthcare [13]. Systematic reviews of mindfulness-based interventions suggest benefits for depression, anxiety, and stress and as an adjunct in treatment of physical health conditions such as cancer and cardiovascular disease [14, 15].

Moreover, MBCT programs have thus far demonstrated lowering of psychological distress [16], depression, and self-reported stress levels in medical students [17]. Recently a systematic review also supports the positive effects of various mindfulness-based interventions in healthcare students, demonstrating benefits in mood, self-efficacy, and empathy [18].

## Research aims

This study is a quality improvement project which assessed the feasibility of a larger scale study such as a full randomised controlled trial (RCT) of a mindfulness intervention among trainee UK hospital doctors to reduce stress. The RE-AIM framework is a framework that has been widely employed to structure such assessments of feasibility in health-related interventions and was therefore used to describe this study [19]. It comprises of five key dimensions including Reach, Adoption, Effectiveness, Implementation, and Maintenance. In this way, RE-AIM focuses on essential programme elements in order to improve intervention reporting. Four of the five dimensions of RE-AIM were used excluding maintenance because this is a short feasibility study. These dimensions were:

- Reach, by establishing the proportion of trainee doctors who expressed an interest to take part in the study;
- Adoption, by identifying the number of participants successfully recruited to participate in the feasibility study;
- Effectiveness (potential effectiveness at this feasibility stage), by establishing whether a difference in the levels of stress, self-reported mindfulness, well-being, working life, and patient relationships over the 6-week period might be reported by the participating trainee doctors;
- Implementation, based on the number of trainee doctors who participated in a minimum of four sessions, participants’ evaluations of the course including modality, logistics, and possible ways to improve attendance;
- Maintenance (not covered in this study).

## Method

### Design

This is a feasibility uncontrolled before-after study which evaluates the effects of a mindfulness course on self-reported levels of stress among foundation junior doctors. In terms of methodology, the primary measure of stress is measured quantitatively but the subjective experiences of participating doctors were also investigated through free-text responses in order to gather valuable feedback and comments to better inform potential further work.

### Setting

This was a single-site study taking place in one university teaching hospital. The trust comprises of 108 foundation doctors made up of 54 first year (F1) and 54 second year doctors (F2). This course only recruited from one trust site as the funding only allowed for one trust to be included in this initial study.

### Eligibility

The eligibility criteria for participating in the mindfulness course were (a) being a foundation junior doctor in the hospital and (b) being able to participate in at least four of the six course sessions. The reasoning was that a minimum attendance and level of engagement was deemed necessary in order to gain benefit from the course. The recruitment target was 20 participants in keeping with the capacity of the course lead.

### Intervention

The intervention was a mindfulness course which was delivered by Breathworks, a secular social enterprise who provide paid-for courses as well as teacher training [20]. Breathworks operate in accordance with the UK Good Practice Guidelines for mindfulness teachers [21]. This 6-week course was an adaptation of the ‘Mindfulness in the Workplace’ course that usually runs over 8 weeks. Participants were provided six 2-h mindfulness sessions between October to December 2017. The content of the course is detailed below (Table 1).

**Table 1:** Course content

2-h session	Exploration of key concepts
	Short guided meditation practices
	Guided mindful activities
	Setting a home practice with a written handout and email with info and link to guided practice
	Discussion of participant experiences of home practice
Home practice	Regular daily practice. Encouraged to aim for 10 min twice daily
	Mindfulness in action tasks set each week
Course handbook	‘The Little Mindfulness Workbook’ [22], accompanied the course and each week’s themes

Knowing that Foundation trainees are notoriously busy, often working an evening on-call rota, a shorter course was opted for so that doctors were more likely to be able to attend all sessions. For the same reason, the time of 17:30 on a weekday after work was chosen so that foundation year (FY) trainees could go straight to the course in the hospital education centre after work. An additional step was to plan the dates of the six sessions to all fall within one rotation of the FY trainee cycle (4-monthly), before changeover day on 5 December 2017.

### Recruitment and procedure

Several months prior to recruitment onto the mindfulness course intervention, two introductory mindfulness sample sessions took place during mandatory teaching time for the F1s and F2s respectively after agreement with the foundation programme director at the trust. These sessions explored the theory and evidence for mindfulness, as well as involving some guided meditation practice, led by a mindfulness teacher from Breathworks. In order to obtain a place on the course, all FY trainees at the trust were invited to respond to an open invitation sent out by email in September 2017 and the places were allocated on a first come, first served basis. However, an equal number of places were reserved for F1 and F2 trainees.

Data collection was undertaken using pre-course questionnaires which were handed out before the first session of the course, and then participants were given post-course questionnaires to fill out at the end of the last session. The data from the questionnaires were distributed and analysed by two members of the team (CB and EC). Questionnaires were completed anonymously. For those who were not present at the first or last session, they were contacted separately and asked to return their completed feedback forms through the education centre department to maintain anonymity.

### Measures

The primary outcome measure was self-reported stress levels measured immediately before and after the course. A one-item self-reported Likert scale was developed for the purposes of this study: ‘Please rate your general levels of stress at work on an average day using the scale below with 0 being completely stress free and 10 being extremely stressed’. Four linked questions were asked at post-intervention (but not at baseline stage) to explore the impact of the mindfulness course on participants’ self-perceived levels of stress: Has the Mindfulness in the Workplace course (i) made you feel less stressed? (ii) improved your ability to recognise when you are feeling stressed? (iii) made you feel better able to understand stress? (iv) taught you skills/techniques to deal with stress? Possible answers were yes, no, and no change.

In terms of secondary measures, the post-course questionnaire also explored the subjective experiences of doctors participating in the mindfulness course with regard to four domains including self-reported mindfulness, its impact on their wellbeing, impact on working life and impact on relationships with patients. Each of these four items had a quantitative aspect where participants were asked if they noticed an improvement in this domain after the course (yes or no answers). Each item also invited any comments (free-text space was provided) for each of these domains.

Attendance and engagement was monitored using a sign-in register at each session. Feedback was also sought on the questionnaire whether the course delivery suited the doctors in terms of timing and length of sessions, as well as format (face-to-face versus online).

### Analyses

No changes were made to the dimensional assessments after study commencement. The RE-AIM framework dimensions were utilised as follows.

#### Reach

The reach of the intervention was assessed by how many FY doctors from the total cohort expressed an interest in taking part on the course multiplied by 100%.

#### Adoption

The degree of adoption was assessed by how many doctors were successfully recruited onto the intervention in total and for FY1 and FY2.

#### Effectiveness

Effectiveness was related to the success rate of the intervention based on predefined outcomes within the confines of the study, which was explored by calculating the effect size for the primary outcome measure of stress reduction. A Mann-Witney *U* test was conducted to analyse the pre-course and post-course scores on the primary ‘effectiveness’ measure. This study was not powered to test any hypotheses, and the purpose of evaluating potential effectiveness was to serve as a preliminary check to observe whether participants tended to be less stressed at the end of the intervention. Descriptive statistics of the participants’ responses (number and percentages of participants) on the linked questions and secondary outcomes are reported in the results. Free-text responses of the participants in the secondary outcomes are presented as quotes in tables.

As any foundation doctors at the trust were eligible to participate, it is possible that doctors in more acute distress may have signed up to the course which might not have been appropriate due to the requirement for more formal healthcare input. Over the duration of the course, all participants were monitored by the course lead and also invited to contact the course lead at any point if they experienced any difficulties that arose, as well as being signposted to the hospital health and wellbeing service which is staffed by appropriate health professionals. The questionnaires also included free space text which allowed participants to report any additional negative effects from the course.

#### Implementation

Implementation was measured firstly by attendance on the course. Descriptive statistics are also provided which assess the implementation potential of the intervention, through participant feedback about course format, delivery, and logistics.

#### Maintenance

No longer-term follow-up was undertaken for this study, and so the fifth dimension of RE-AIM maintenance could not be evaluated.

## Results

### Reach

**Fig. 1:**
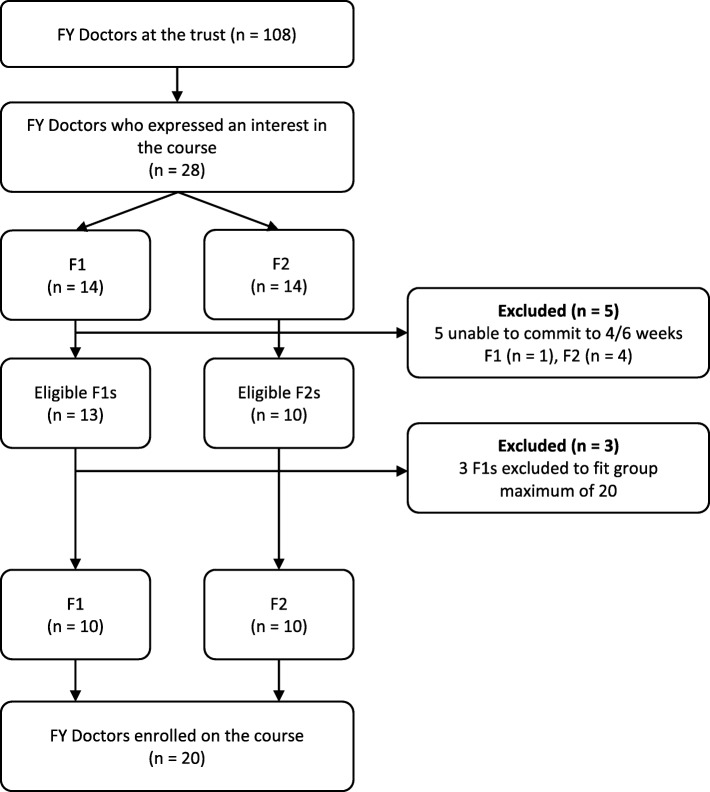
Participant flow through the study

Of the 108 FY doctors at the trust, 28 expressed an interest in participating in the course (26.0%); however, due to the maximum of 20 places available, the percentage recruited onto the course was as expected at 18.5%. Five eligible FY participants were excluded as they were unable to commit to at least 4 weeks, and three F1 doctors were unable to take part as all places had been filled (see Fig. 1).

### Adoption

All 20 places on the course were filled during recruitment, with 10 F1s and 10 F2s. Of these 20, 10 were female and 10 were male.

### Effectiveness

#### Questionnaire completion

The pre-course questionnaire pack was completed by 16 out of 20 participants (80%), and the post-course questionnaire was completed by 14 out of 20 participants (70%).

#### Primary outcome measure-perceived stress

The median baseline stress score of the participants was 6.5 (range = 2 to 9). The median post-course stress score was 5.0 (range = 2 to 8). The Mann-Witney test indicated that the stress levels of participants were significantly lower at the end of the course compared to baseline, *U* = 74.50, *p* = .04.

In terms of the four linked questions which were administered at post-intervention only, 71% of participants who completed the post-course questionnaire reported that they felt less stressed as a result of the course. 100% of participants reported that they were better at recognising and managing stress, 93% of participants reported that they understand stress better as a result of the course, and 100% of participants felt that the course had taught them skills/techniques to better deal with stress.

#### Secondary outcomes

All participants reported that they were more mindful and had improved overall wellbeing. In terms of overall wellbeing, doctors mentioned the course made them realise it is ‘okay to take time out for self’, ‘reduced anxiety’, and ‘encouraged me to take time out of the day and check in with my own sense of wellbeing’. Another reported ‘relaxation techniques at home have really helped me switch off and sleep’, and one doctor stated the course had a ‘positive impact on my thought process’. Twelve out of 14 participants reported that the mindfulness course had a positive effect on their working life and their relationship with patients, whereas two participants reported no change on these aspects. Regarding working life, doctors reported that they find it easier to ‘prioritise jobs’ and ‘concentrate better’, and they felt they were ‘more of a pleasure to work with’. For their relationships with patients, they also reported they were ‘more understanding of difficult patients’, ‘more mindful of what they are going through’, ‘more patient’, and ‘more compassionate’. Some examples of qualitative responses of the participants in these questions are presented in Table 2.

**Table 2:** Secondary measures

	Yes (%)	No	Illustrative quotations on the impact of the course
Feel more mindful	14 (100%)	0	‘I enjoy day to day tasks more and appreciate things more’ ‘Can focus on the task in hand’ ‘Able to recognise when being mindful’
Improved overall wellbeing	14 (100%)	0	‘Reduced my anxiety. Kinder to self’ ‘The course helped me realise it’s okay to time out for self’ ‘Feel happier’ ‘Encouraged me to take time out of the day and check in with my own sense of wellbeing’ ‘Relaxation techniques at home have really helped me switch off and sleep’
Positive impact on working life	12 (86%)	2	‘I am more of a pleasure to work with’ ‘Prioritise jobs better’ ‘Can concentrate better’ ‘I am less stressed by understanding when and why I am stressed, and stop negative cycle of stress and inefficiency’ ‘Too early to have effect, but in the long term will have effects’
Positive effect on patient-doctor relationship	12 (86%)	2	‘I am more mindful of what they are going through… less likely to react’ ‘More understanding of difficult patients’ ‘I think about how patients feel, and be kinder to self and them’ ‘More compassionate, seeing from their perspective’ ‘Be mindful of interactions with patients to improve their hospital stay’ ‘The main issue with patients is time - this course not impacted that’ ‘More patient’

In terms of assessing harms, there were no negative responses for any of the items on the questionnaire.

### Implementation

Sixteen participants (80%) attended one or more session. The mean sessions completed by the participants were 2.45 out of the 6-week course. The number of participants who attended at least 4 of the 6 sessions was 7 out of 20 (35%). The main reason for non-attendance was work commitments followed by personal reasons, annual leave, and demands of other courses. We asked participants some additional questions about acceptability and delivery of the intervention which might be important aspects of the implementation potential. All 14 respondents would recommend the course to another foundation doctor, with one doctor stating ‘you won’t regret it… reminds you to look after you’.

In terms of delivery, 13 participants felt a face-face approach would be more beneficial than an online course and 1 participant did not answer the question. They qualified their answer by explaining that they valued the open discussion of a small group, and it was useful to share ideas and experiences. For example, they said, ‘it needs to be in a group’ due to there being ‘so many different experiences’ and it was ‘good to meet together and share …it normalises feelings’. All doctors felt a 6-week programme was an appropriate course length, and the majority (*n* = 13) stated the 2-hour sessions were suitable. One participant suggested it could have been reduced to 1–1.5 h as they found it ‘hard to concentrate’. All felt the number of participants was appropriate as well as the course location.

## Discussion

The aim of this study was to evaluate the feasibility of a larger evaluation and implementation of the ‘Mindfulness in the Workplace course’ for FY doctors. Based on the evaluations of four (reach, effectiveness, adoption, and implementation) out of the five dimensions of the RE-AIM framework, the findings suggest that further development and testing are required prior to trialling the intervention programme in a larger-scale study such as a randomised controlled trial.

In terms of the reach and adoption of the study, 28 doctors across a total of 100 eligible doctors expressed an interest in participating in the study. This number exceeded the 20 places on the course, and so all places were successfully filled, demonstrating a degree of successful adoption. The sample session, which gave all FY doctors some brief exposure to mindfulness during mandatory teaching time prior to recruitment, may have played a key role in accounting for the reach achieved in this study, but this link is not directly evidenced from our evaluation and further work should enquire more about these factors that increased likelihood to participate. An RCT at a multi-site level would benefit from more information about ways to maximise reach across sites. More demographic information such as gender, age, ethnicity of both participants, and population would also help assess the representativeness of enrolled participants. The limited funding, lack of population demographics, and single-site nature of this study limited the assessment of both reach and adoption.

Although the intervention was targeted at all FY doctors, it is difficult to know whether enrolled participants were the most or least stressed FY doctors in the population. It is possible that FY doctors who are in need of stress reduction were unable to attend due to their stress, and the least stressed FY doctors of the cohort subsequently participated in the course. Equally those who did not enrol might have felt that they had insufficient stress for the intervention. Participants from a qualitative study looking at perceptions of GP resilience training suggested that those most likely to benefit from resilience training were the least likely to engage due to stress and time pressure mitigating against their engagement [23]. This idea is a potentially crucial barrier to reach and needs to be explored more. One suggestion to investigate this would be to survey the whole FY doctor cohort (participants and non-participants) to compare baseline stress levels.

This study, as an early stage quality improvement project, did not aim to rigorously assess effectiveness. For this reason, we considered it appropriate to use a non-validated stress measure which was devised for the purposes of the study. A statistically significant reduction in self-reported stress was observed at the end of the intervention. However, this study was not powered to detect statistically significant differences in participant stress levels before and after the course with this small sample size. This is an encouraging finding for larger future evaluations of mindfulness courses in FY doctors, and importantly, there were no negative responses to any of the questionnaire items. The linked questions and qualitative responses of participants suggest that the intervention may lead to important benefits for doctors. However, a major limitation of the study was the lack of validated measures which limits its usefulness as a feasibility study to inform an RCT. Further studies would benefit from using validated outcome measures to assess the levels of stress and wellbeing in trainee doctors. Such measures could be burnout (e.g. Maslach Burnout Inventory [24]) and quality of life. Overall, the small sample size combined with the use of brief non-validated measures in this feasibility study offers limited information about the health benefits in response to this intervention. A larger RCT would benefit from a prior specified sample size to adequately assess effectiveness using validated measures.

It is also worth noting that of the 20 FY participants who signed up to the course, only 80% completed the pre-course questionnaire pack and only 70% completed the post-course pack. These completion rates are generally satisfactory compared to the completion rates reported by a recent systematic review which evaluated completion rates of 14 international mindfulness interventions in doctors and health providers [25]. However, it may be that those who attended the course were more likely to complete the pack because they were allocated time during the first and last sessions of the course to complete it. For the others contacted by email, there may not be enough incentive to respond. Junior doctors are often bombarded with various surveys regarding their clinical and educational activities, impacting on the likelihood of completing yet another survey. Further work might ask FY doctors to physically complete a paper survey during mandatory cohort teaching time to increase response rate prior to the intervention taking place. Additionally this strategy could also be used for the follow-up in place of emails.

Regarding implementation, 16 participants (80%) managed to attend 1 or more sessions but this leaves 4 (20%) having signed up and not come to any sessions. The number of participants who received the full intervention course (4 of the 6 sessions) was low (*n* = 7; 35%). The low completion rate in this study also echoes a recent qualitative study among UK doctors [23]. The most common reason for non-attendance was due to ‘work commitments’. This is particularly concerning if doctors are missing out on possible benefits due to organisational work constraints such as rotas or on-calls. This term ‘work commitments’ may encompass a variety of reasons for non-attendance, and further work should explore more detail into what exactly prevented them from attending. These might be avoidable absences, which could potentially be addressed in further work to improve implementation. Poor engagement was anticipated from the outset, and the project therefore included specific design features such as having a mandatory sample session, taking place at work, opting for a shorter course, and planning dates within a job rotation. The course was also planned immediately after working hours, but doctors might justifiably struggle to volunteer any further time after a busy day. Feedback about the course delivery and logistics was positive which supports enquiry into these other potential barriers.

Alternative formats such as applying institutional time-protected schemes to attend the course for trainee doctors would perhaps improve the completion rate. Indeed, trials which applied institutional financial support and time-protected schemes have shown promising completion rates and enhanced effectiveness in the international literature [26]. The North West of England Foundation School supported the mindfulness sample sessions during mandatory teaching time, and this use of mandatory time could potentially help overcome problems of implementation. Additionally, future work might investigate alternative methods of delivering mindfulness such as brief ward-based sessions or short practices integrated into teaching sessions. Online forms of mindfulness do also exist, but the feedback from this study suggested that FY doctors felt additional value in meeting up in person with other FY doctors.

Finally, although this study has provided some useful information for the first four of the five RE-AIM dimensions, it failed to report on the dimension of maintenance and whether any such promising benefits of the course might be sustained in the following weeks and months after course completion. It will be important to address this fifth arm in future work to evaluate any longer-term impacts. This could be addressed by repeating the same measures at set time points after the course has finished.

## Strengths and limitations

The study’s major limitation was the use of non-validated brief measures of stress and wellbeing. Additionally, the sample size was small, limited demographic information was collected, and there was no follow-up to assess maintenance. The lack of control group and randomisation process means that the possibility for participation bias cannot be excluded. However, this study was intended as a quality improvement project by junior doctors for junior doctors at a single site supported by limited institutional funds. In terms of the strengths, participants were satisfied with the delivery components of the course and positive feedback was obtained regarding benefits with parallel absence of any reported harms. Furthermore, there is little in the literature that studies a mindfulness intervention in FY doctors and so this study provides valuable information to guide future RCTs in the UK.

## Conclusion

This is the first programme of research to evaluate the feasibility of trialling a modified ‘Mindfulness at Work’ intervention for junior hospital doctors in the UK. This study developed our understanding of key aspects of the success of a future RCT including reach, adoption, effectiveness, and implementation. Based on the findings from this study, we conclude that this mindfulness course is promising but further modifications are required before this intervention programme may be trialled and implemented in the UK. More intermediary work with elements closer to an RCT design including an evaluation at multiple sites, the use of validated effectiveness measures, and a control arm is a valid way forward. Moreover, our findings suggest that improving the engagement of doctors is a core area where more support is needed, and further qualitative research could inform new ways to increase doctor engagement with the programme.

## Abbreviations

F1: Foundation year 1 doctor
F2: Foundation year 2 doctor
FY: Foundation year
GMC: General Medical Council
MBCT: Mindfulness-based cognitive therapy
RCT: Randomised controlled trial
